# Comparison of Upper Central Incisor Torque in the ClinCheck^®^ with and without CBCT Integration: A Cross-Sectional Study

**DOI:** 10.3390/dj12080269

**Published:** 2024-08-20

**Authors:** Cíntia Queirós, Maria Gonçalves, Sofia Ferreira, Inês de Castro, Rui M. S. Azevedo, Teresa Pinho

**Affiliations:** 1 Oral Pathology and Rehabilitation Research Unit (UNIPRO), University Institute of Health Science (IUCS), CESPU, 4585-116 Gandra, Portugal; a28163@alunos.cespu.pt (C.Q.); a28293@alunos.cespu.pt (S.F.); a28233@alunos.cespu.pt (I.d.C.); 2 Associate Laboratory i4HB—Institute for Health and Bioeconomy, University Institute of Health Sciences—CESPU, 4585-116 Gandra, Portugal; mprazeres.goncalves@iucs.cespu.pt (M.G.); rui.azevedo@iucs.cespu.pt (R.M.S.A.); 3 UCIBIO—Applied Molecular Biosciences Unit, Translational Toxicology Research Laboratory, University Institute of Health Sciences (1H-TOXRUN, IUCS—CESPU), 4585-116 Gandra, Portugal; 4 UCIBIO—Applied Molecular Biosciences Unit, Forensics and Biomedical Sciences Research Laboratory, University Institute of Health Sciences (1H-TOXRUN, IUCS—CESPU), 4585-116 Gandra, Portugal

**Keywords:** torque, orthodontic appliances, removable, cone beam computer tomography, tooth movement techniques

## Abstract

Controlling root movement is one of the greatest challenges in orthodontic treatment with aligners, like Invisalign^®^ aligners. Cone Beam Computed Tomography (CBCT) integration into ClinCheck^®^, enabling bone and root visualisation, allows a more accurate follow-up of the teeth position. This study aims to compare torque measurements of the upper central incisors with and without CBCT and relate them to the upper incisor inclination and facial biotype. In a sample of 70 teeth, torque measurements were obtained by importing images into AutoCAD^®^ software (version 2024). The angle between the tooth’s long axis with CBCT duplicate and the tooth’s long axis without CBCT was obtained to assess the difference. Statistically significant differences between torque measurements with and without CBCT were found, as well as between these measurements and the inclination of the upper incisors. No statistically significant differences were found among the facial biotypes. The average values of 27.8° ± 3.4° and 21.5° ± 3.2° were obtained for the angle between the axes. Torque without CBCT was lower than torque with CBCT, for the same tooth. The angle between the axes had a similar mean for both teeth. CBCT integration into ClinCheck^®^ allows for a more correct torque measurement.

## 1. Introduction

Over the years, the field of orthodontics has been revolutionised by the technological advances that have emerged and that have contributed significantly to improving diagnosis and treatment planning [1,2]. The quest to meet patients’ needs, both in terms of aesthetics and comfort, led to the appearance of Clear Aligner Therapy (CAT) as an alternative to conventional bracket treatment [3,4]. Besides aligners, there are fixed and aesthetic alternatives to conventional brackets, such as ceramic brackets and lingual brackets. However, the lingual bracket system is a technique that involves a lot of manual dexterity, making it one of the most difficult to perform, while ceramic brackets have superior failure rates compared to conventional brackets [5,6]. A study conducted by Pascoal et al. assessed the relationship between the personality traits of laypeople and the aesthetic perception of different orthodontic appliances. They reported that aligners were the most frequently chosen, followed by aesthetic brackets and finally metal brackets, with neuroticism being associated with the choice for conventional orthodontic treatment [7].

Orthodontic movement consists of moving the tooth within the alveolar bone with the bone being an important factor in limiting such movements [8]. Therefore, the orthodontist has to respect the limits of the bone surrounding the dental piece, since excessive orthodontic movements and loss of torque can lead to root resorption, gingival recession and loss of alveolar bone, resulting in deteriorated periodontal conditions [9,10].

According to Andrews, one of the keys of normal occlusion is torque, which represents the buccolingual inclination of the teeth’s crown [11]. An adequate torque value of the anterior teeth is extremely important as it not only provides a stable and ideal occlusal relationship, with adequate overjet and overbite, but also has a positive influence on the aesthetics of the smile and the profile of the soft tissues [9,12,13]. Furthermore, an appropriate torque value provides correct anterior guidance and gives the clinician satisfactory final results [9].

Controlling root movement is one of the greatest challenges in orthodontic treatment with aligners, like Invisalign^®^ aligners. Cone Beam Computed Tomography (CBCT) integration into ClinCheck^®^ Pro^®^ 6.0 software has enabled bone and root visualisation, facilitating treatment planning and providing a more accurate reflection of the patient’s final teeth position. In addition, CBCT has made it possible to guarantee root parallelism and to prevent fenestrations and dehiscence [14,15].

Since complex cases require three-dimensional (3D) planning in order to control movements, a proper understanding of CBCT and its integration into ClinCheck^®^ is essential. This understanding helps to prevent periodontal problems by providing the orthodontist with an image of the surrounding alveolar bone, allowing them to realise the limitations of movement, avoiding the appearance of bone defects such as dehiscence and fenestrations as well as root resorptions [9].

The objective of this study was to compare the torque measurements of the upper central incisors without and with CBCT integration, and to measure the angle resulting from the intersection of the long axis of the tooth in the case without CBCT with the duplicate of the long axis of the tooth with CBCT for both teeth. Additionally, this study also aimed to relate the torque measurements with upper central incisor inclination and facial biotype. In this way, at a clinical level, it will help the orthodontist to ascertain whether all cases require CBCT as a complement to ClinCheck^®^ or whether CBCT only applies to the most complex cases, to help the measurements of the incisor torque on additional aligners. It also aims to provide orthodontists with an understanding of whether they can simply use clinical crowns as a reference for longitudinal assessment.

Therefore, we define the following null hypotheses: H0: coronal torque measurements of maxillary central incisors without CBCT integration do not differ significantly from the coronal–root torque measurements with CBCT integration; H0: the angle measurements do not show consistent means; H0: the differences between torque measurements of maxillary central incisors without and with CBCT integration are not influenced by maxillary central incisor inclination; H0: the differences between torque measurements of maxillary central incisors without and with CBCT integration are not influenced by the facial biotype; and H0: coronal torque measurements without CBCT do not differ significantly from coronal–root torque measurements with CBCT for all types of maxillary central incisor inclination and facial biotype.

## 2. Materials and Methods

### 2.1. Study Design

This research employed a cross-sectional design. Patient data were collected during a specific period to compare measurements with and without CBCT integration.

### 2.2. Participants, Eligibility Criteria, and Settings

The sample consisted of 35 participants with permanent dentition (with and without third molars), who visited Doctor Teresa Pinho’s (Specialist in Orthodontics and Invisalign^®^ Diamond Provider) private clinic in São João da Madeira, Portugal. Our final sample included 70 teeth (35 upper right incisors and 35 upper left incisors) that were measured 140 times in total (with and without CBCT for each tooth), providing us with a considerable number of measurements to analyse.

Participants had to fulfil certain criteria in order to be included in this study, including the following: permanent dentition, without attrition on the crowns of the upper central incisors, CBCT radiographs already taken and integrated into the ClinCheck^®^ Pro^®^ 6.0 software, and complete and available cephalometric analyses. Patients who did not meet these criteria were excluded. In addition, informed consent was obtained from all the patients in the study when they entered the clinic, where they consented that their photographic and radiographic records, which were taken for diagnostic and clinical control purposes, could be used in research work, articles, and scientific presentations. Their confidentiality was always guaranteed.

This research is part of a dissertation project that, as it involves data from patients from a private clinic, required approval by the Ethics Committee of the University Institute of Health Sciences (IUCS-CESPU), under reference 11/CE-IUCS/2024.

### 2.3. Interventions

Data collection instruments

- Intra-oral scanner (Itero^®^ Element 5D Plus, Align Technology, Tempe, AZ, USA);
- Itero^®^ software version 1.34.0.3 (Align Technology, Tempe, AZ, USA);
- CBCT files;
- ClinCheck Pro^®^ 6.0 software (AlignTech, Santa Monica, CA, USA);
- Orthodontic and clinical patient reports.

Data processing instruments

- Microsoft Excel 2016 version;
- AutoCAD^®^ software version 2024 (Autodesk, Inc., San Rafael, CA, USA);
- IBM Statistical Program for Social Sciences—SPSS^®^, software version 29.0.

Procedures regarding data collection

Firstly, demographic characteristics like age at the beginning of the treatment and gender were collected from the 35 patients who met the inclusion criteria. The convenience sample was built with patients selected sequentially within a timeframe.

Next, images with and without CBCT integration from the Invisalign^®^ Doctor Site (IDS) programme, with the same teeth inclination, were collected. Right and left lateral perspectives were gathered resulting in 4 images of each patient and 2 of each tooth.

Lastly, orthodontic reports were examined to collect cephalometric data such as overjet, overbite, type of inclination of the upper central incisors, interincisal angle, and facial biotype. The facial biotype and upper central incisor inclination were collected in order to analyse the relation with the torque measurements obtained from the ClinCheck^®^. Regarding the type of inclination of the upper central incisor, it has a normal reference value of 22° ± 2° (normal inclination). If the value is higher than the norm, the incisors are proclined, and if lower than the norm, retroclined [16]. In this study, we only collected the type of inclination and not the value.

Measurements

**Figure 1:**
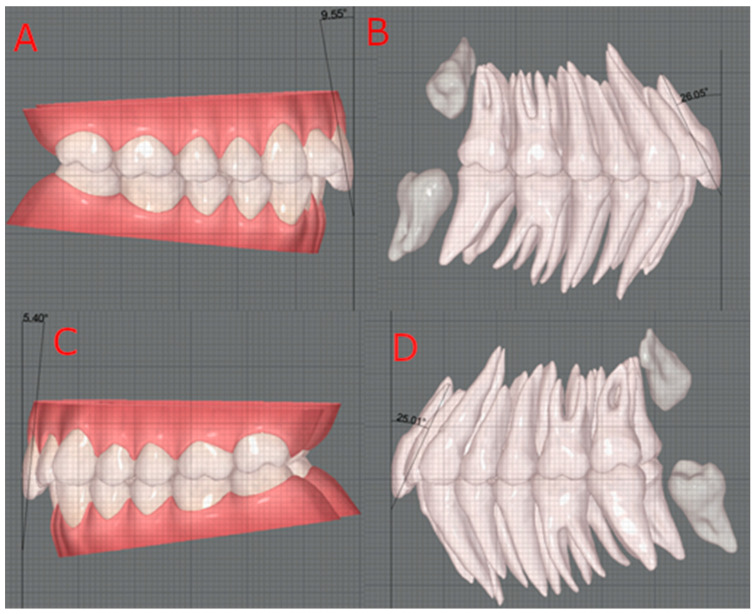
Torque measurement: right upper incisor without Cone Beam Computed Tomography (CBCT) (**A**); right upper incisor with CBCT (**B**); left upper incisor without CBCT (**C**); left upper incisor with CBCT (**D**).

Based on the study by Zuñiga et al., these images were imported into AutoCAD^®^ software, and the measurements were taken using 2 decimals [17]. Then, a true vertical line was traced adjacent to the tooth surface in all the images. Next, in the cases without CBCT integration, the most incisal point of the incisal edge and the most gingival point of the crown of the tooth were joined, forming the long axis. In the cases with CBCT integration, the most gingival point was replaced by the most apical point of the upper central incisor’s root. The angle between the two lines was measured to determine torque. These steps were executed on the images with and without CBCT integration in both upper central incisors (Figure 1).

In cases without CBCT, we referred to the torque as coronal torque because the long axis of the tooth was represented only by the crown. In the cases with CBCT, the name was changed to coronal–root torque as the long axis of the tooth was formed by connecting a coronal point to a root point.

**Figure 2:**
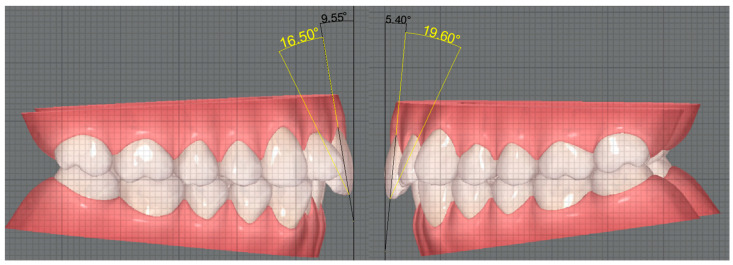
Angle resulting from the intersection of the long axis of the crown and the duplicate of the long axis of the tooth.

After the torque measurements, we proceeded to measure the angle between the long axis of the tooth with CBCT and the long axis of the tooth without CBCT in the 70 teeth of the sample. The long axis of the tooth was duplicated in the CBCT cases. Then, the duplicate was moved to the image without CBCT, where the incisal reference point of the tooth was used to position the line, thus intersecting the long axis of the crown. Finally, the angle resulting from this intersection was measured (Figure 2).

### 2.4. Statistical Analysis

The analysis of the data was carried out using IBM^®^ SPSS software (Statistical Program for Social Sciences), version 29.0 for Windows. Descriptive statistics were produced to provide estimates of frequencies and percentages, means, medians, standard deviations, minimums, and maximums. The Shapiro–Wilk test was used to assess the normality of the sample, with no evidence of rejection of the null hypotheses.

Data normality led us to use the one-sample t-test to compare torque measurements of upper central incisors with and without CBCT integration. In order to assess the agreement between the two torque measurement methods, with and without CBCT, we used the Bland–Altman plot method.

The intraclass correlation coefficient (ICC) was used to analyse the reliability of inter- and intra-operator measurements in order to assess whether there is absolute agreement between the results of the two examiners, whereby ICC of 0.0–0.1, poor agreement; 0.01–0.2, slight agreement; 0.41–0.6, moderate agreement; 0.81–0.99, almost perfect agreement; and 1.0, perfect agreement [18].

To compare torque measurements of upper central incisors with and without CBCT integration, according to the inclination of upper central incisors and facial biotype, one-way ANOVA was used, followed by the Bonferroni test. The effect sizes for the ANOVA were determined using η^2^ values, with the thresholds considered being η^2^ = 0.01 for a small effect, η^2^ = 0.06 for a medium effect, and η^2^ = 0.14 for a large effect. The significance level was set at 0.05.

## 3. Results

### 3.1. Baseline Data

**Figure 3:**
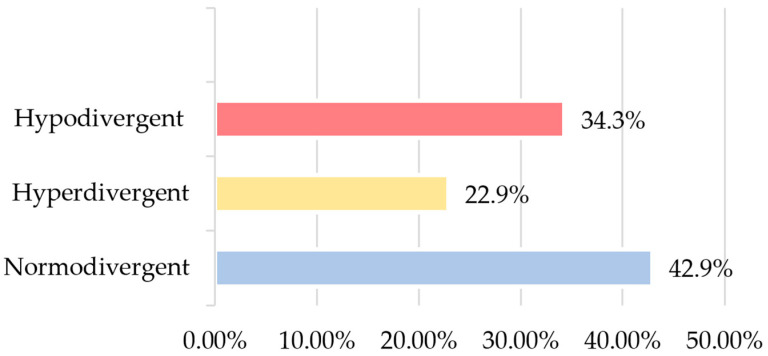
Sample distribution according to facial biotype.

Considering the inclusion criteria, our final sample consisted of 35 participants (70 upper central incisors), with ages ranging from 13 to 71 (mean = 32.0; SD = 16.9), of whom 24 (68.6%) were female and 11 (31.4%) were male. In terms of cephalometric data relating to facial biotype, 15 (42.9%) were normodivergent; 12 (34.3%), hypodivergent; and 8, (22.9%) hyperdivergent (Figure 3).

**Figure 4:**
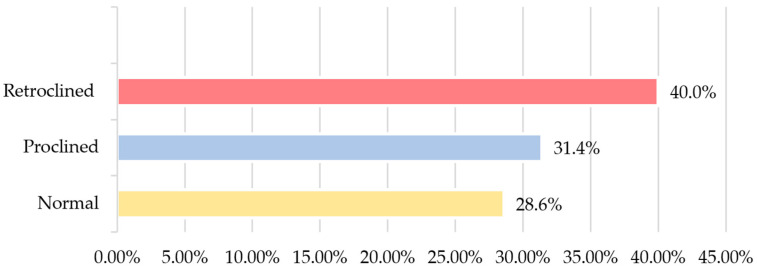
Sample distribution according to upper incisor inclination.

Among the total cephalometric sample, 25 (71.4%) patients presented normal overjet, and 18 (51.4%) showed an increased overbite. With regard to the inclination of the upper central incisors, 14 (40.0%) had retroclined incisors (Figure 4). In relation to the interincisal angle, 37.1% had a normal angle, 34.3% had an increased angle, and 28.6% had a decreased angle.

### 3.2. Comparison of Coronal Torque Measurements without CBCT and Coronal–Root Torque Measurements with CBCT

Comparing intra-operator measurements of the coronal torque of the central incisor 11 without CBCT to those of the coronal–root torque with CBCT, statistically significant differences were found (t (34) = −38.5; *p* < 0.001), with the coronal–root torque measurements showing higher values. Likewise, the coronal torque measurements of the central incisor 21 without CBCT revealed significantly lower values compared to the coronal–root torque measurements with CBCT (t (34) = −38.3; *p* < 0.001) (Table 1).

The inter-operator measurements follow the same direction, with significantly higher coronal–root torque values for upper central incisors 11 (t (34) = −38.2; *p* < 0.001) and 21 (t (34) = −38.7; *p* < 0.001) (Table 1).

#### Inter- and Intra-Operator Analysis

Inter- and intra-operator reliability were assessed using the intraclass correlation coefficient (Table 2). The ICC results were 0.999 for coronal torque 11 and 21, respectively, revealing almost perfect reliability/concordance between the two evaluators.

The agreement between the two evaluators showed a high degree of agreement with R squared values of 0.998 for tooth 11 and 0.997 for tooth 21.

### 3.3. Angle Resulting from the Intersection of the Long Axis of the Tooth without CBCT and the Duplicate of the Long Axis of the Tooth with CBCT

Table 3 shows the mean values and standard deviation of the angle resulting from the intersection of the long axis without CBCT and the duplicate of the long axis with CBCT for both incisors (Table 3).

### 3.4. Comparison of Coronal Torque Measurements without CBCT with Coronal–Root Torque Measurements with CBCT, According to the Inclination of Upper Central Incisors

The mean values of the coronal torque and the coronal–root torque separated by the classes of upper central incisor inclination obtained by cephalometry are shown in Table 4. We found statistically significant differences in the mean values of coronal torque 11 between the three types of inclination (F (2, 32) = 13.5; *p* < 0.001), with these differences being between incisors with normal inclination (3.2° ± 3.7°) and retroclined incisors (−3.7° ± 6.9°) (*p* = 0.01), and between retroclined and proclined incisors (7.1° ± 3.8°) (*p* < 0.001). The mean coronal–root torque 11 values were also different between normal inclined (25.5° ± 3.3°) and retroclined (18.4° ± 6.8°) incisors (*p* = 0.011) and between retroclined and proclined incisors (28.1° ± 5.0°) (*p* < 0.001), with proclined incisors exhibiting higher average coronal–root torque values.

As for coronal torque 21, proclined incisors were the ones that had the highest mean torque values (8.9° ± 4.3°), with statistically significant differences between these and those with retroclination (2.0° ± 6.9°) (*p* < 0.001) and between incisors with normal inclination (4.6° ± 3.9°) and retroclination (*p* = 0.049). In coronal–root torque 21, there were only statistically significant differences between proclined (30.2° ± 5.1°) and retroclined (20.5° ± 7.1°) incisors (*p* < 0.001) (Table 4).

### 3.5. Comparison of Coronal Torque Measurements without CBCT with Coronal–Root Torque Measurements with CBCT, According to the Facial Biotype

With regard to the mean values of the coronal torque and the coronal–radicular torque of the upper central incisors according to the facial biotype obtained by cephalometry, in all measurements, hypodivergent individuals had higher values than normodivergent and hyperdivergent individuals, respectively, but these differences were not statistically significant.

## 4. Discussion

A proper buccolingual inclination of the upper central incisor is extremely important during orthodontic treatment, as it plays a crucial role in both smile aesthetics and occlusal functionality [13,14,19].

With the incorporation of CBCT, the orthodontist has more detailed information compared to two-dimensional (2D) radiographs, with considerably less radiation than the conventional computed tomography radiographs. In addition, diagnosis and treatment planning are facilitated because CBCT gives the clinician specific 3D models of each patient’s mouth structure. Its integration is not exclusive to Invisalign^®^, with CBCT being integrated into other aligner systems. Thus, it is possible to know not only the sizes of the anatomical structures including teeth, but also to obtain precise measurements such as the coronal–root torque [20,21]. Nevertheless, there is always a risk associated with exposure to ionising radiation, especially in younger patients. The routine use of CBCT in orthodontics is not recommended according to international guidelines. As a rule, it is prescribed in surgical cases, dental abnormalities such as impacted teeth and dentofacial deformities [22].

So far, no study has compared torque measurements with and without CBCT in the ClinCheck^®^ software to assess the influence of its integration on torque measurements. Therefore, we collected images from the IDS programme with the same teeth inclination, with and without CBCT, that is, with and without the presence of tooth roots, respectively. The images were imported into AutoCAD^®^ software and the measurements taken using two decimals [17]. Next, adapting the true vertical line used in the Castroflorio et al. study, we drew it tangent to the buccal surface of the tooth [23]. For the occlusal plane, it did not need to be drawn since the images taken from the software already showed the maxilla and mandible in occlusion. To trace the long axis of the tooth in cases without CBCT, we used the method already followed by Tepedino et al., using the most incisal and gingival points as reference [4]. Meanwhile, in CBCT cases, the most gingival point was replaced with the most apical point of the root, a reference used in many studies [9,10,11,15,20].

Various current articles on torque measurement in ClinCheck^®^ have evaluated tooth movement only through differences in the dental crown, as they did not analyse the integration of the CBCT into the software [2,4,13,23]. Consequently, the long axis of the crown represents, in this case, the long axis of the tooth. However, the long axis of the crown often does not correspond exactly to the long axis of the tooth due to its morphology [19]. Generally, the upper central incisor has a convex rather than flat labial surface, which means that when connecting the most gingival point with the most incisal point to trace the long axis of the crown, it can often not coincide with the long axis of the tooth, which uses the most apical point of the root and the most incisal point of the crown [24].

Since torque is highly affected by anatomical variations and tooth morphology, it might be expected that its measurement would differ when measured, on the same tooth, using both the long tooth axis and the long coronal axis [25].

Using inferential statistics, our study revealed that the measurements of coronal torque without CBCT differed significantly (*p* < 0.001) from the coronal–root torque measurements with CBCT, for both the right and left upper central incisor. For both cases, the torque without CBCT proved to be lower than the torque with CBCT. Thus, the null hypothesis was rejected. This can be explained by the fact that both measurements were made using different reference points, resulting in this discrepancy in values. This proves that CBCT is indeed an asset in ascertaining torque, since the presence of the crown alone can be misleading. However, one cannot conclude that it is wrong to use the axis of the crown as the long axis of the tooth. Even if these axes do not coincide, using the crown may be sufficient during orthodontic treatment, without subjecting the patient to amounts of radiation from the CBCT just to visualise the root. Therefore, the long axis of the tooth in the case with CBCT was duplicated and transposed to the case without CBCT. The angle between the duplicate and the long axis of the tooth without CBCT was obtained in the 70 teeth. Using descriptive statistics, we obtained average values of 27.8° ± 3.4° for the right central incisor and average values of 21.5° ± 3.2° for the left incisor. These results show that the means are consistent between the two teeth, therefore rejecting the null hypothesis. A study carried out by Zuñiga et al. obtained average angle values of 21.34° ± 4.41° for the upper incisors, similar to our values. However, this study did not use the most apical point as a reference, reporting that through not using this point, a more accurate representation of the long axis of the root is obtained. Since there are variations in the position of the root apex, this method avoids problems in measuring the angle. Even so, the average values in this study were similar to our results, proving that both methods are effective for assessing the angle [17].

Fredericks, on the other hand, used a methodology more similar to ours to obtain the long axis of the tooth, using the apical and incisal points as references, obtaining average angle values of 23.88° for the upper incisors, very close to our values [26]. In addition to differences in the methodology of tracing the long axis of the tooth, these studies also traced the long axis of the crown differently, opting for the line tangent to the buccal surface of the tooth, while we opted for the line interlocking the most incisal and most gingival point of the crown. Taking into account the oscillations in the incisal–gingival curvature of the crown and the variations in the position of the apex among the upper central incisors, it is to be expected to have seen these differences in the angle values between the studies [17,26]. Nevertheless, the values were all in the same range, proving the viability of all the measurement methods mentioned above, including ours.

The crown–root angle means remain stable over time, making longitudinal crown studies a reliable method for assessing treatment effects, as validated by Oliveira et al. [13]. This approach supports the use of crown assessment to monitor long-term orthodontic progress, together with the complementary role of CBCT in the early phases of treatment, where it can be used as a powerful diagnosis tool. CBCT provides crucial 3D images for accurate diagnosis and treatment planning, particularly in complex cases, ensuring a precise placement of the initial aligner. Then, subsequent additional aligners can be guided with crown–root angle adjustments, minimising the need for further CBCT scans, thus reducing the patient’s radiation exposure and maintaining treatment accuracy.

For our study, we collected the inclinations of the upper incisors provided by cephalometric radiographs, which were given by the angle formed by the long axis of the tooth (apex to tip of the incisor) and the NA line (line from point N to point A) [25].

We found statistically significant differences in the mean values of coronal torque 11 between the three types of incisor inclination, with the average coronal torque values always being higher in proclined incisors. Likewise, coronal torque 21 and coronal–root torques 11 and 21 were all greater in the proclined incisors. Meanwhile, the lowest torque values were found in retroclined incisors, both in cases with and without CBCT, for both incisors. Thus, the null hypothesis was rejected. These results are in line with the findings of two articles that divided the torque according to the direction of movement. Oliveira et al. and Gaddam et al. divided the sample into proclination movement when it was necessary to buccalise and retroclination when it was necessary to lingualise the tooth [13,14]. This can help clinically by providing the orthodontists with more accurate information about the buccolingual inclination of the tooth, so that they can apply torque more precisely during treatment.

In a study conducted by Kuc-Michalska et al., the authors reported that when the inclination of the tooth increased, the density of the buccal alveolar bone also increased, but the density of the palatal bone decreased. The opposite occurred when tooth inclination decreased, leading to a reduction in buccal alveolar bone. This can be explained by the fact that when the tooth is programmed to move, the crown follows one direction while the root follows the opposing direction [26]. In addition, Mirmohamadsadeghi et al. reported in their study a decrease in the apical bone in upper central incisors with proclination [9]. Tian et al. reported an increase in fenestrations when the incisors were retroclined due to the low thickness of the alveolar bone on the buccal side, while the proclined incisors had a greater thickness of buccal alveolar bone. The authors therefore advise for the early detection of bone defects that pre-exist, orthodontic treatment, and the application of mild forces during orthodontic movements in order to reduce bone loss [11].

Our results show that in normal, proclined, or retroclined incisors, for the same tooth, the torque was significantly higher when measured with CBCT integration compared to when measured without CBCT. In this way, the null hypothesis was rejected. Considering these differences in torque, we must emphasise that the presence of CBCT can be valuable in the treatment of more complex cases because the clinician can see the tooth as a whole and not just the clinical crow, thus preventing the appearance of bone defects [11,27].

There are three types of vertical facial growth patterns. In the hyperdivergent growth pattern, the growth vector is vertical, resulting in a more posterior chin position, which contrasts with the hypodivergent growth pattern, where the vector is horizontal. In turn, the normodivergent pattern has a downward and forward growth [28]. That being said, hyperdivergent individuals tend to have longer faces and retroclined upper incisors, while hypodivergent individuals appear to have shorter faces and proclined upper incisors [29]. In accordance with these data, our results show that hypodivergent individuals had both higher coronal and coronal–root torque values compared to hyperdivergent individuals, both for the right and left incisors. However, there were no statistically significant differences, so the null hypothesis was not rejected. This could be explained by the fact that the difference between measuring torque simply with the crown or with the crown and root using CBCT is indifferent to the facial biotype. Additionally, the majority of the population studied has a normodivergent facial biotype (42.9%), with hyperdivergent patients being the minority. One possible reason for this difference may be due to the majority of the clinic’s patients having a normodivergent facial biotype and, in a sample of 35 patients, such proportions are to be expected. To ensure a more balanced sample, future studies with more patients could help to uniformise the sample in terms of facial biotype and contribute to these results. In addition, facial biotype is closely related to the type of facial musculature, as it has been reported that in hyperdivergent growth, patients tend to have muscle hypofunction, while the same is not observed in the hypodivergent type. The same applies in terms of alveolar bone, with the findings of Sadek et al. and Gaffuri et al. showing that hyperdivergent patients have a thinner alveolar bone compared to hypodivergent individuals, suggesting a reduction in incisor tooth movements during orthodontic treatment in hyperdivergent cases [30,31].

Once again, we observed that, regardless of facial biotype, torque was significantly lower when measured without CBCT compared to when measured with CBCT integration. Hence, the null hypothesis was rejected. These findings prove what was mentioned above, demonstrating that CBCT, being incorporated into the software, provides a more accurate measurement of the tooth’s buccolingual inclination, thereby giving the clinician more security when dealing with complex hyperdivergent cases, where movements have to be smaller and more cautious to prevent bone defects [30].

## 5. Conclusions

The coronal torque without CBCT was always lower than the coronal–root torque with CBCT, for the same tooth, in all the measurements taken.

The mean torque values were always higher when the measurements were taken with CBCT compared to when they were taken without CBCT, regardless of the type of inclination as well as the facial biotype.

The integration of CBCT into the ClinCheck^®^ software allows for a more accurate measurement of tooth torque. In addition, CBCT is extremely valuable in the diagnosis phase, particularly in complex cases. However, the use of the crown remains a correct and effective approach to monitoring the long-term progress during the orthodontic treatment (with additional aligners).

## Figures and Tables

**Table 1:** Comparison of upper central incisor torque without and with Cone Beam Computed Tomography (CBCT).

	Test Value = 0		
Intra-Operator	Mean Differences (°)	*t*	*p*
Coronal torque 11—coronal–root torque 11	−21.9	−38.5	<0.001
Coronal torque 21—coronal–root torque 21	−27.4	−38.3	<0.001
Inter-operator			
Coronal torque 11—coronal–root torque 11	−21.7	−38.2	<0.001
Coronal torque 21—coronal–root torque 21	−21.2	−38.7	<0.001

**Table 2:** Intraclass correlation coefficient (ICC).

	ICC	CI 95%
Torque 11	0.999	[0.998–0.999]
Torque 21	0.999	[0.997–0.999]

**Table 3:** Descriptive statistics of the angle resulting from the intersection of the two axes.

	N	Mean (°)	SD (°)	Minimum	Maximum
Angle 11	35	27.8	3.4	15.2	28.1
Angle 21	35	21.5	3.2	14.9	27.7

Angle 11: angle for tooth 11; Angle 21: angle for tooth 21.

**Table 4:** Coronal torque and coronal–root torque of upper central incisors, according to the inclination of the upper central incisors.

Measurement	Inclination of Central Incisors	N	Mean ± SD (°)	*p* Overall	*p* NvsPI	*p* NvsRT	*p* PIvsRT	Effect Size
Coronal torque 11	Normal	10	3.2 ± 3.7	<0.001				
	Proclined	11	7.1 ± 3.8		ns	0.01	<0.001	0.17–0.61
	Retroclined	14	−3.7 ± 6.9					
Coronal–root torque 11	Normal	10	25.5 ± 3.3	<0.001				
	Proclined	11	28.1 ± 5.0		ns	0.011	<0.001	0.12–0.57
	Retroclined	14	18.4 ± 6.8					
Coronal torque 21	Normal	10	4.6 ± 3.9	<0.001				
	Proclined	11	8.9 ± 4.3		ns	0.049	<0.001	0.12–0.57
	Retroclined	14	2.0 ± 6.9					
Coronal–root torque 21	Normal	10	25.7 ± 3.7	<0.001				
	Proclined	11	30.2 ± 5.1		ns	ns	<0.001	0.09–0.54
	Retroclined	14	20.5 ± 7.1					
11 CBCT-wCBCT	Normal	10	22.1 ± 3.0	ns				0.01–0.16
	Proclined	11	21.0 ± 3.5					
	Retroinclined	14	22.1 ± 3.6					
21 CBCT-wCBCT	Normal	10	21.1 ± 2.5	ns				0.01–0.21
	Proclined	11	21.3 ± 3.0					
	Retroinclined	14	18.6 ± 8.3					

ns: *p* > 0.05; N—normal inclination (22° ± 2°); PI—proclined (>24°); RT—retroclined (<20°); CBCT—with CBCT; wCBCT—without CBCT.

## Data Availability

Data supporting the findings of this study are available from the corresponding author upon reasonable request.
